# Associations between malaria and local and global climate variability in five regions in Papua New Guinea

**DOI:** 10.1186/s41182-016-0021-x

**Published:** 2016-08-04

**Authors:** Chisato Imai, Hae-Kwan Cheong, Ho Kim, Yasushi Honda, Jin-Hee Eum, Clara T. Kim, Jin Seob Kim, Yoonhee Kim, Swadhin K. Behera, Mohd Nasir Hassan, Joshua Nealon, Hyenmi Chung, Masahiro Hashizume

**Affiliations:** 1 School of Public Health and Social Work, Queensland University of Technology, 60 Musk Avenue, Brisbane, 4064 QLD Australia; 2 Department of Pediatric Infectious Diseases, Institute of Tropical Medicine, Nagasaki University, 1-12-4 Sakamoto, Nagasaki, 852-8523 Japan; 3 Department of Social and Preventive Medicine, Sungkyunkwan University School of Medicine, 300 Cheoncheon-dong, Jangan-gu, Suwon, Gyeonggi-do 440-746 Republic of Korea; 4 Department of Biostatistics, Graduate School of Public Health, Seoul National University, 599 Gwanak-ro, Gwanak-gu, Seoul, Republic of Korea; 5 Faculty of Health and Sport Sciences, The University of Tsukuba, Comprehensive Research Building D 709, 1-1-1 Tennoudai, Tsukuba, Japan; 6 Japan Agency for Marine-Earth Science and Technology (JAMSTEC), Yokohama Institute for Earth Science, 3173-25 Showa-machi, Kanazawa-ku, Yokohama, 236-0001 Japan; 7 World Health Organization Western Pacific Regional Office, P.O. Box 2932, 1000 Manila, Philippines; 8 National Institute of Environmental Research, Hwangyong-ro 42, Seogu, Incheon Republic of Korea

**Keywords:** Malaria, Weather, Climate, Papua New Guinea, Climate change

## Abstract

**Background:**

Malaria is a significant public health issue in Papua New Guinea (PNG) as the burden is among the highest in Asia and the Pacific region. Though PNG’s vulnerability to climate change and sensitivity of malaria mosquitoes to weather are well-documented, there are few in-depth epidemiological studies conducted on the potential impacts of climate on malaria incidence in the country.

**Methods:**

This study explored what and how local weather and global climate variability impact on malaria incidence in five regions of PNG. Time series methods were applied to evaluate the associations of malaria incidence with weather and climate factors, respectively. Local weather factors including precipitation and temperature and global climate phenomena such as El Niño-Southern Oscillation (ENSO), the ENSO Modoki, the Southern Annular Mode, and the Indian Ocean Dipole were considered in analyses.

**Results:**

The results showed that malaria incidence was associated with local weather factors in most regions but at the different lag times and in directions. Meanwhile, there were trends in associations with global climate factors by geographical locations of study sites.

**Conclusions:**

Overall heterogeneous associations suggest the importance of location-specific approaches in PNG not only for further investigations but also public health interventions in repose to the potential impacts arising from climate change.

**Electronic supplementary material:**

The online version of this article (doi:10.1186/s41182-016-0021-x) contains supplementary material, which is available to authorized users.

## Background

Papua New Guinea (PNG) is a malaria endemic country where all four human *Plasmodium* species (*Plasmodium falciparum*, *Plasmodium vivax*, *Plasmodium malariae*, and *Plasmodium ovale*) circulate in the population with varying distribution and degrees of endemicity [1]. In the nation, malaria is the leading cause of outpatient visits, the fourth leading cause of hospital admissions, and the third most common cause of death [2]. Despite significant reductions of malaria morbidity and mortality in many Pacific and Asian countries, the disease remains a serious public health issue in PNG, and instead, a localized increase in malaria prevalence has been reported over recent decades in the country [3]. Surveys in the 1940s and 1950s showed no cases in highland region, but began to report malaria from the 1960s [4, 5]. One of the possible contributors for the localized increase is global warming as the changes in the disease distribution and intensity of transmission have been witnessed following warming temperatures in other highland areas around the globe [6–8].

As a coastal country lying in the tropical Pacific Ocean, PNG is regarded highly vulnerable to the effects of climate change. In fact, rising sea levels and warming trends in both annual and seasonal mean air temperatures have already been reported for Port Moresby [9]. Given the nation’s vulnerability to climate change and high public health burdens of malaria, it is important for the country to gain proper understanding about the potential impacts of climate change on the infectious disease in order to prepare them with integrated action and strategic malaria control and prevention programs. To understand the impacts of climate change, firstly, it is critical to know the current condition of associations between malaria and climate. However, in PNG, there is limited information only available from descriptive assessments, and few epidemiological studies have ever focused on the topic in depth. The present study was therefore developed to investigate how and what local weather and global climate variability are associated with malaria among different regions in PNG.

Local weather factors of our interest in this study include rainfall and temperature. The dependence of malaria transmission on those weather factors is a well-accepted fact due to their significant roles in population dynamics of mosquito vectors. Generally, a minimal volume of rainfall is essential to create the water pools necessary for vector breeding and larval habitats, and minimum ambient temperatures are required below which mosquito vectors, and parasites within them, are biologically unable to develop [5, 10]. In a preceding study, temperature is described as the primary determinant of malaria incidence as endemicity is dependent on altitude on which temperatures also depend [11].

As the extent of local weather factors, global climate variability was also taken into consideration in the present study. Global climate here refers to ocean and atmosphere phenomena such as El Niño-Southern Oscillation (ENSO). Because precipitation and temperature conditions are linked to ocean-atmosphere phenomena, the potential indirect impacts of ocean-atmosphere phenomena on malaria transmission in the other parts of the world have been documented elsewhere [12–15]. In PNG, local weather has a very significant relationship with sea surface temperature (SST) as the average monthly air temperature and year-to-year variability in rainfall are highly impacted by ENSO [9]. One extreme example for the impacts of ocean environment on the weather in PNG is the episode of a severe drought caused by the strong El Niño event in 1997 [16]. Considering those relationships between local weather and global climate variability, global climate seems more likely to indirectly influence malaria transmission in PNG through the local weather factors.

For global climate variability, not only ENSO but also the ENSO Modoki, the Southern Annular Mode (SAM), and the Indian Ocean Dipole (IOD) were considered in the present study. The El Niño Modoki is a coupled ocean-atmosphere phenomenon in the tropical Pacific that is different from the canonical coupled phenomenon, El Niño. El Niño is characterized by abnormally warmer SST in the eastern tropical Pacific than usual, whereas El Niño Modoki is characterized by anomalous warming in the central Pacific and anomalous cooling in the eastern and western Pacific [17]. In terms of climatic influence, however, both extreme El Niño and El Niño Modoki events impact in a similar manner on PNG since SST in the western Pacific becomes unusually cooler and brings low rainfall to the country during those events [9, 16]. Data describing the effects of IOD on PNG weather are scarce. However, a typical IOD event is characterized by cooler SST in the eastern part of the Indian Ocean near Indonesia and often results in a decrease of precipitation in the neighboring country, Australia [18]. The effects of SAM on Australia are similarly well-documented while little is known in PNG [19, 20]. With a view to improve understanding of global climatic determinants of malaria incidence in geographically distinct foci of PNG, a comprehensive assessment of these ocean-atmospheric phenomena was performed.

## Methods

### Malaria and local weather data

Data on monthly malaria cases from 1996 to 2008 were obtained from the National Health Information System of PNG. Four administrative provinces and one district were included in this study. They were the southern part of Western province, Eastern Highlands province, East Sepik province, Madang province, and Port Moresby. All study locations were located in the coastal lowlands, except for Eastern Highlands province. Western province and Port Moresby are located at the southern coastal area, and East Sepik and Madang provinces are on the northern coast. Eastern Highlands province is landlocked and in a mountainous region (>1600 m above sea level). Data on local weather factors (e.g., precipitation, minimum and maximum temperature) were acquired from the PNG National Weather Service.

### Ocean climate data

The events of ENSO were represented by NINO3.4 anomaly index, defined by the anomaly from the SST climatology of 1981–2010 in the NINO3.4 region (5° N–5° S, 170–120° W) of the Pacific Ocean. SAM, also known as the Antarctic Oscillation, refers to the alternating pattern of strengthening and weakening westerly winds with high and low pressure bands between the mid and high latitudes in the Southern Hemisphere [19]. Its index was defined by the monthly mean 700-hPa height anomalies at 20° S which was projected onto the leading Empirical Orthogonal Function of monthly 700-hPa 1979–2000 data [21]. The evolution of IOD is represented by the dipole mode index (DMI), defined as the difference in SST between the western (10° S–10° N, 50–70° E) and eastern (10° S–0°, 90–110° E) tropical Indian Ocean [22]. The data for those three climate indices and the measurements described above were obtained from the U.S. National Oceanic and Atmospheric Administration (NOAA). The El Niño Modoki index (EMI) was defined with SST anomaly by 1982–2010 base period as

1 $$ \mathrm{E}\mathrm{M}\mathrm{I}={\mathrm{SSTA}}_{\mathrm{central}}-0.5\ \left({\mathrm{SSTA}}_{\mathrm{east}}\right)-0.5\ \left({\mathrm{SSTA}}_{\mathrm{west}}\right) $$

where SSTA indicates sea surface temperature anomaly of the area mean regions specified as the central (165° E–140° W, 10° S–10° N), the east (110°–70° W, 15° S–5° N), and the west (125°–145° E, 10° S–20° N) [23]. The data for calculating the index was obtained from Japan Agency for Marine-Earth Science and Technology (JAMSTEC). The examined period of time for the respective local weather and global climate models of each study location is described in the supplemental material (Additional file 1: Table S1).

### Statistical analysis

A time series method was applied to evaluate the associations of malaria incidence with local weather and global climate variability, respectively. Two-step approaches were applied. A generalized additive model, which flexibly models nonlinearity with smoothing splines [24], was initially used to visualize the responses of exposure factors to malaria incidence, since little is known in prior about the relationships. We then confirmed the linear relationships with weather and climate variability and generalized linear models (GLMs) with negative binomial distribution were used to estimate the associations. The distribution selection for GLM is described in the section of sensitivity analysis.

Local weather model:

2 $$ \log \left({Y}_t\right)={\beta}_0+{\beta}_1{\mathrm{temperature}}_{t-l}+{\beta}_2{\mathrm{rain}}_{t-l}+\mathrm{ns}(t)+ \log \left(\mathrm{population}\right) $$

Global climate model:

3 $$ \log \left({Y}_t\right)={\beta}_0+{\beta}_1{\mathrm{EMI}}_{t-l}+{\beta}_2\mathrm{NINO}3.4{\mathrm{Anom}}_{t-l}+{\beta}_3{\mathrm{SAM}}_{t-l}+{\beta}_4{\mathrm{DMI}}_{t-l}+\mathrm{ns}(t)+ \log \left(\mathrm{population}\right) $$

The outcome number of reported malaria cases at month *t*, denoted by *Yt*, was considered as an outcome variable while predictor variables were the local weather and global climate. In the local weather model, minimum temperature instead of maximum temperature was included due to better data completeness. The ns(*t*) denotes the natural cubic spline function on the observational time to remove seasonality variations of each location in respective local weather and global climate models. The optimal degrees of freedom for the natural cubic spline on the observational time were selected based upon the lowest Akaike’s Information Criterion (AIC). The *t* − *l* for each weather and climate variable of interest denotes a lag time. Lags for local weather models were designed as moving averages from the month of the event (0 month) to 3 months prior (0–1, 0–2, and 0–3-month average). The global climate models in turn included the moving average from the month of the event (0 month) to 6 months in prior (0–1, 0–2, 0–3, 0–4, 0–5, and 0–6-month average). Those lengths of lag times were first determined a priori based on biological plausibility and then assessed by cross correlation functions to confirm no obvious discrepancy with a priori approach (Additional file 1: Figure S1 and S2). The examined period of time for the respective local weather and global climate models of each study location is described in the supplemental material (Additional file 1: Table S1). The annual population of each study site was included with the offset function. The statistical analyses were performed with the statistical software package R version 2.15.3 [25].

### Sensitivity analysis

As alternative methods for controlling for seasonality, calendar time variables of month and year, the periodic harmonic functions called Fourier series formed by the sum of sines and cosines, and natural cubic splines for observational time were compared by AIC. The lowest AIC were observed with the model with natural cubic splines, and consequently, the method was chosen for seasonal control. The distribution selections for GLM, whether Poisson or negative binomial, were also examined by overdispersion parameters. The result was that the parameter proved the overdispersion of all models with Poisson distribution, and they were improved by using GLM negative binomial distribution models.

## Results

### Local weather factors

**Fig. 1:**
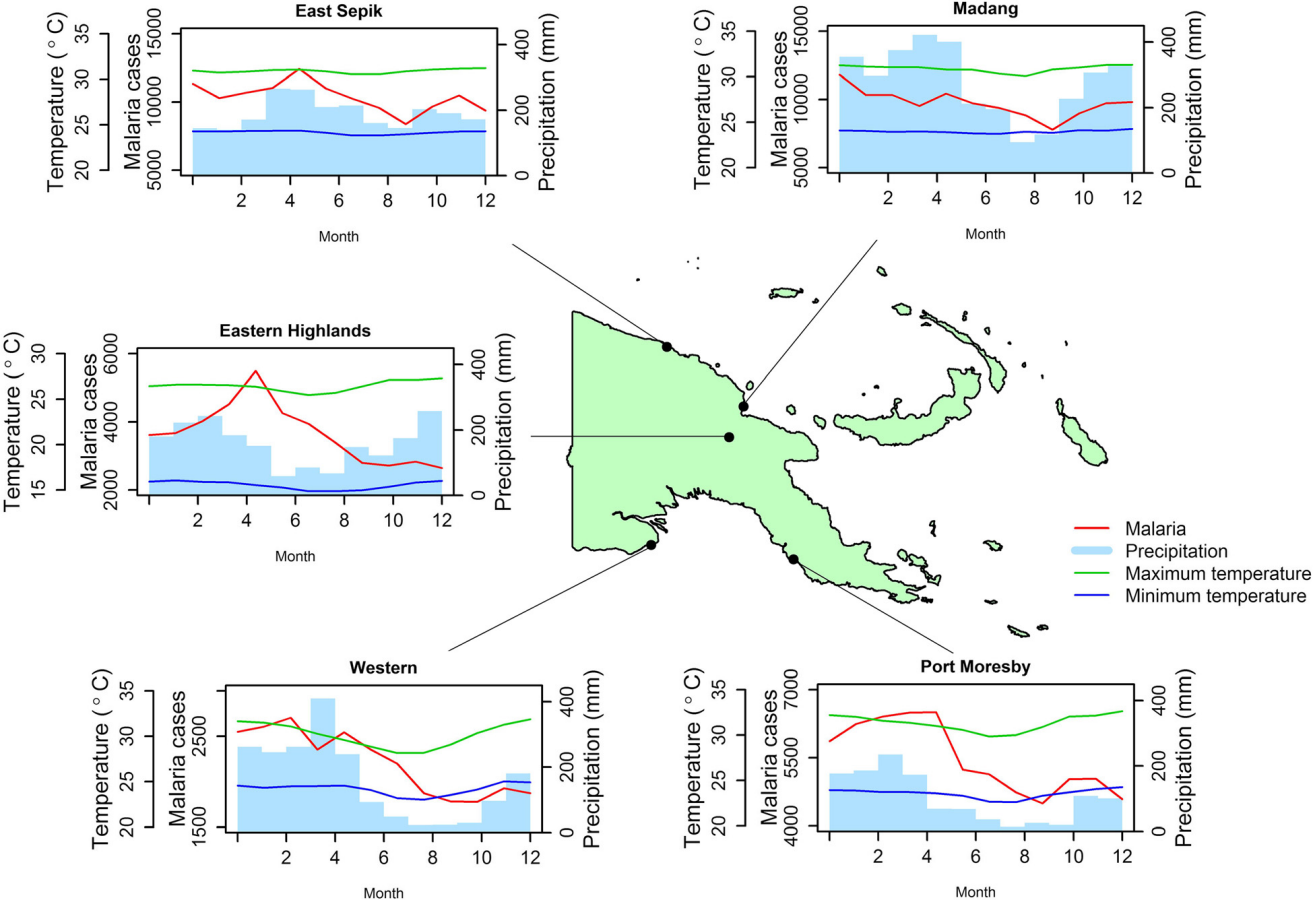
Study locations and seasonal variations of malaria cases and weather factors. The locations of five study sites in Papua New Guinea are shown with the monthly averages of malaria reported cases, precipitation (mm), and maximum and minimum temperatures (°C)

Figure 1 shows the locations of study sites and their monthly average of malaria reported cases, precipitation, and temperature. There is minimal seasonal variation in temperatures, but it shows distinct seasonal patterns in precipitation and reported malaria cases in each region of PNG. The seasonality of monthly precipitation seems to coincide with malaria cases, with the notable exception of Eastern Highlands. The other distinctive characteristic of Eastern Highlands is the low temperature, since the province is located at high altitude. Time series plots for malaria cases and local weather factors during the study period are also available in the supplemental material (Additional file 1: Figure S3).

**Fig. 2:**
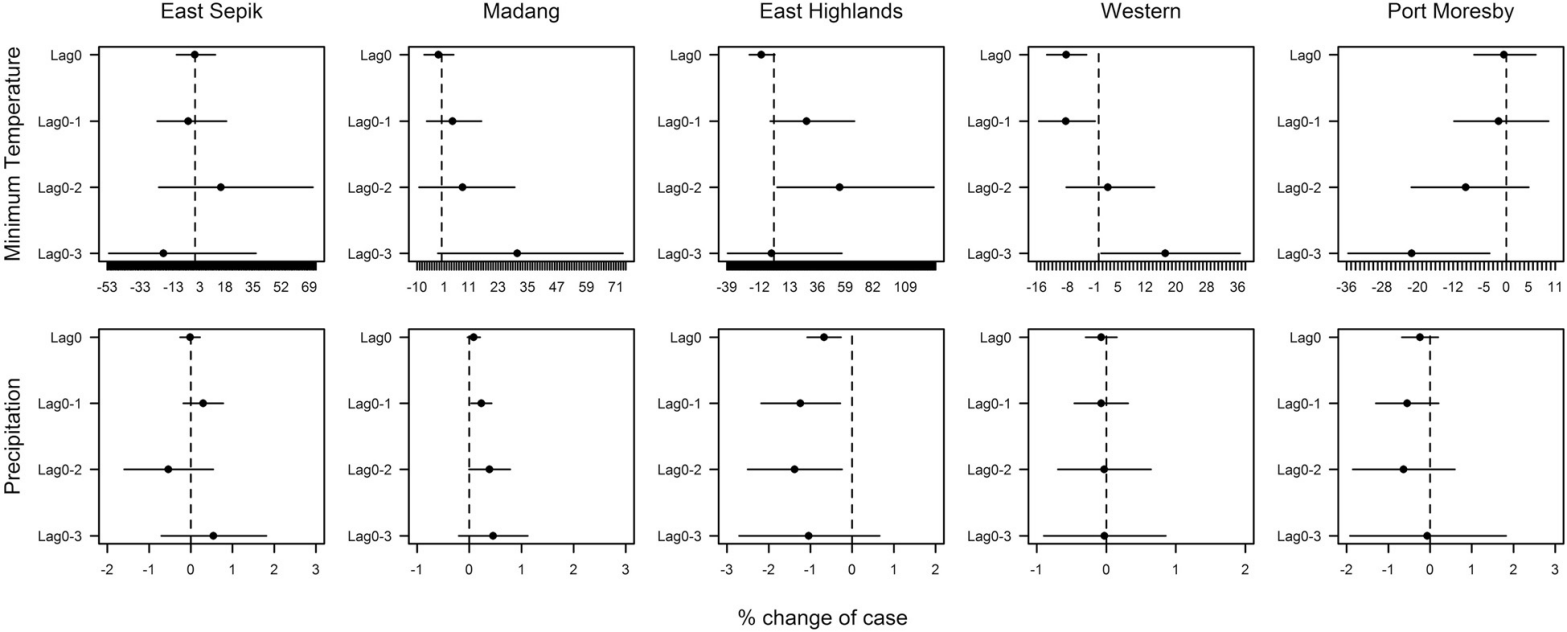
The percentage change of malaria cases with 1 °C temperature and 10 mm precipitation increment. The graphs show the effects of temperature and precipitation at different lag times on malaria cases in the five study locations. The effects are indicated by the percentage changes of the number of malaria cases with 1 °C minimum temperature and 10 mm precipitation increase. The *dots* and *bars* are the estimates and 95 % confidence intervals of the percent change

The results of time series analysis varied across study locations (Fig. 2). Malaria incidence in Western province was significantly negatively associated with minimum temperature at the current month and 1 month before (0–1 month), yet the direction of the association shifted to positive from lags of 2 months (0–2 months). At the lag of 2 months, minimum temperature was not significantly associated but it later became significant with an increased strength of the association at the lag of 3 months (0–3 months). Although the exact strength of the associations differed by lags, the transitions in direction of the association from negative to positive with minimum temperature were similarly observed in Eastern Highlands and Madang. In Eastern Highlands, the impact was initially observed negatively at the current month and then shifted towards positive with a lag of 1 month and the 2-months lag which recorded a significant association. Madang, in turn, did not provide any significant relationships at any lags, yet the association altered from negative to positive with an increase in strength over time. The contrast was Port Moresby where the strength of negative associations grew over lag times, and the association was significant with a lag of 3 months. In East Sepik, no notable associations with minimum temperature were observed.

For precipitation, a pronounced trend in associations was displayed only in Eastern Highlands and Madang. In Eastern Highlands, the significantly negative associations were found at current and at lags of 1 and 2 months. In contrast, for Madang, significant positive associations were observed at 1- and 2-month lag times.

### Global climate factors

**Fig. 3:**
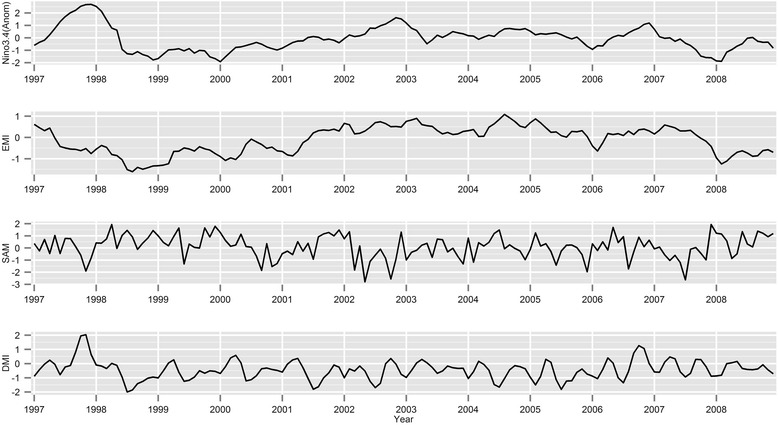
Time series plots for NINO3.4 anomaly, EMI, SAM, and DMI from 1997 to 2008

**Fig. 4:**
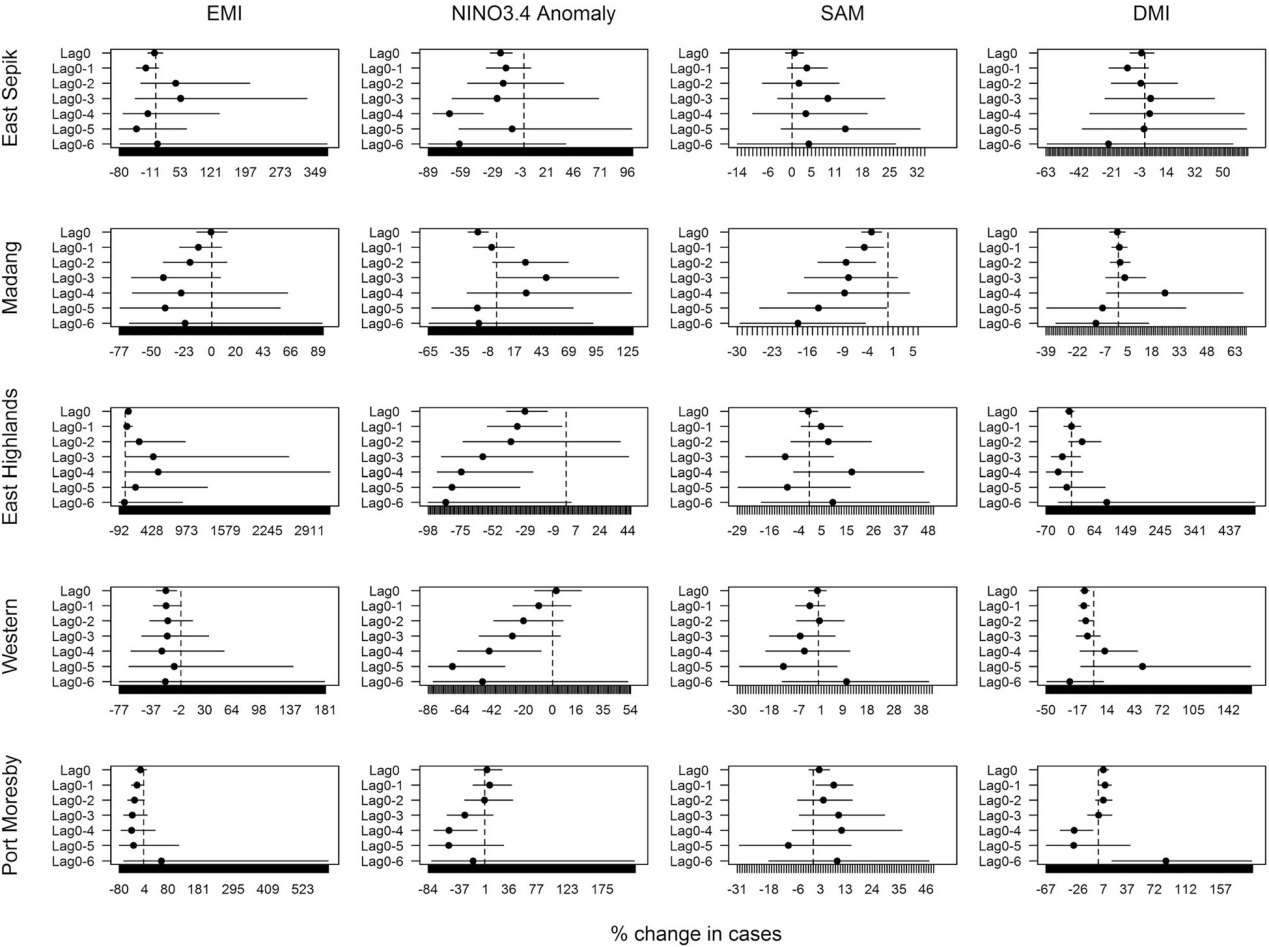
The percentage change of malaria cases with 1 unit increment of global climate indicators. The graphs show the effects of EMI, NINO3.4 anomaly, SAM, and DMI at different lag times on malaria cases in the five study locations. The effects are indicated by the percentage changes of the number of cases with every 1 unit increment change in the global climate indicators

The correlations between global climatic factors at the same month were weak, with the maximum Pearson’s correlation (*r*) to be 0.46 between NINO3.4 anomaly and EMI (Additional file 1: Figure S4). Figure 3 presents the time series of global climate indices from 1997 to 2008. Compared with local weather factors, the effects of global climate had more consistent direction of associations over lag times. There were also trends in association by geographical location of study site (Fig. 4). Table 1 presents a summary of significant associations with their directions and lag times. EMI was negatively associated in the southern coastal locations, Western and Port Moresby, at commonly short lags of the current month (0 month) and 1 month (0–1 month). Despite the absence of significant associations, the results revealed that there was a consistent negative association in Madang. Eastern Highlands, in contrast, was positively associated with EMI. NINO3.4 anomaly had negative associations in all study locations. The northern coastal locations of East Sepik and Madang provinces were more likely to be immediately affected (no lags) by NINO3.4 anomaly whereas the impacts in the southern locations Western and Port Moresby were observed much later, after 4 or 5 months. Eastern Highlands, located in the central mountainous area between the northern and southern coastal locations, had associations in both immediate and later lag times (0, 0–1, 0–4, and 0–5 months). Although the direction of associations was different, malaria incidence in the southern coastal locations, Western and Port Moresby, was significantly associated with DMI. In Western province, malaria cases were negatively associated with DMI from the immediate month to 2 months before DMI. Malaria incidence in Port Moresby was conversely positively related with DMI at the current and 1 month prior, yet shifted to a negative association at 4-month lag (0–4 months). The impact of SAM was the least associated with malaria cases among all global indicators. In Port Moresby, the trend in associations was less consistent but was significant with 1-month lag (0–1 month). Madang was the only town that had consistently negative associations with SAM at both short and long lags.

**Table 1:** The summary of significant associations with global climate index

	Global climate indices							
Region	EMI		NINO3.4 anomaly		SAM		DMI	
	Direction	Lag	Direction	Lag	Direction	Lag	Direction	Lag
East Sepik			−	0, 0–4				
Madang			−	0	−	0, 0–1, 0–2,0–5, 0–6		
			+	0–3				
Eastern Highlands	+	0–2, 0–3, 0–4	−	0, 0–1, 0–4, 0–5				
Western	−	0, 0–1	−	0–4, 0–5			−	0, 0–1, 0–2
Port Moresby	−	0–1, 0–2	−	0–4	+	0–1	+	0, 0–1
							−	0–4

## Discussion

The impacts of temperature and precipitation have been extensively studied as the primary determinants of malaria incidence since they exert critical biological influence on development and life cycle of both mosquito vectors and malaria parasites. In particular, temperature is a fundamental determinant of parasite development, and the length of extrinsic incubation period is highly sensitive to the ambient temperature [5, 10].

In our results, there were negative associations with minimum temperature in three study locations (i.e., Western, Eastern Highlands, and Madang provinces) when the immediate impact is considered (lag 0). However, positive associations were present when 2- to 3-month time lags were introduced. The lead time prior to observable positive effects varied by location, but the time-dependent associations observed in this study agree with previously published studies [26, 27]. The positive association observed at 2- to 3-month lags also seems biologically plausible given the life cycle of *Anopheles* mosquitoes and parasites. Malaria incidence in Port Moresby and East Sepik, on the other hand, had different responses to minimum temperature, showing either null or negative associations. This may suggest that we failed to consider the substantial confounders or needed a more biologically relevant variable for ambient temperature. For instance, Plasmodium development depends on not only minimum temperature but also depends on the diurnal temperature range [28, 29]. This is an indicator that we may consider in future analyses.

Observed relationships between levels of precipitation and malaria also varied by study regions. This is not a surprising finding, considering that the effect of the weather variability can be very location specific. Generally, aquatic reservoirs are essential for mosquito survival and breeding. However, the impacts of different volumes and frequencies of precipitation on entomological and epidemiological parameters may vary substantially from one ecosystem to another. A number of studies support this hypothesis, reporting contradictory findings which suggest that the impacts of precipitation are strongly context dependent [30–35]. For example, rainfall may promote malaria transmission by creating ground pools and other water sources in which vectors can breed, yet heavy rains can have flushing effects, removing these habitats [5]. Drought at where rainfall is normally abundant, on contrary, may eliminate predators and result in safe havens for mosquitoes since mosquitoes are susceptible to a range of vertebrate and invertebrate predators found in the wetlands [36]. Accordingly, malaria epidemics have been reported in the year following a drought in Venezuela [37]. The impacts of rainfall will therefore be location specific and require interpretation of epidemiology and vector ecology of individual sites.

In contrast to results of local weather analysis, global climate indicators revealed more intuitive findings. The extreme positive ENSO and El Niño Modoki events usually link to lower volumes of precipitation in PNG. Likewise, positive IOD events bring lower precipitation in neighboring Australia [18]. Therefore, the negative associations between malaria cases and those global climate indices (ESNO, El Niño Modoki, and IOD) found in this study may reflect the conditions of lower malaria transmission and lower precipitation or vice versa. The reason why this is not completely consistent with the findings with the local weather analysis is uncertain. Addressing the meteorological bases for the relationships between local weather and global climate factors is beyond the scope of this study. One possible explanation, however, is that the ocean exchanges the various components of atmospheric conditions such as heat, water, gases, and air circulations. Global climate indices that encompass those different aspects of local weather may increase the probability of malaria incidence and thus resulted as plausible predictors for the disease in this study.

There are some limitations in this study. Foremost, the assessed outcome was a clinical diagnosis based on the presence and the history of fever. The accuracy of case detection in PNG has been previously described [38]; thus, there is a high possibility that our data includes misclassified cases. This would have been serious issues in terms of proper treatments at the individual level and the precise estimates of impacts. However, clinical diagnosis can be still sufficient to capture the association trends with weather factors as studies showed that suspected cases have the seasonal variations similar to laboratory confirmed cases [39, 40], and the statistical focus of time series is relative changes in cases. In addition, because these relative changes are examined by monthly variations, other changes in much longer time scales (e.g., yearly) do not greatly affect or confound in our analysis. For instance, the changes of the predominant malaria species are reported in PNG [3], but it is very unlikely that those factors rapidly change in months. This applies to other important non-climatic determinants of malaria incidence which include intervention programs, socio-economic development, increased population movements, agriculture, urbanization, and drug resistance. Moreover, if any, potential impacts of such influences were also minimized by adjusting seasonality and long-term trends in our models.

Secondly, there were some missing local weather data (i.e., maximum temperature), particularly from Eastern Highlands. This hampered exploring sensitivity of different measurements of weather variability such as diurnal range of temperature. In addition, this may have created bias in assessment results and reduced the power of analysis due to the shorter observable period of time than that of global climate indicators. The missing data, however, seemed to have occurred randomly (e.g., data from a single month or season was not consistently absent) and is thus likely free of substantial systematic bias (Additional file 1: Figure S4).

## Conclusions

In our study, the local weather and global climate factors that interplayed and highlighted their associations with malaria incidence vary by study sites. This is not a surprising result as substantial heterogeneity of malaria epidemiology due to environmental and cultural diversity in PNG has been also described in the previous study [11]. Certainly, further investigations for better understanding of the topic is necessary, but more importantly, our findings suggested significance of location-specific research and implementation of malaria interventions. The location-specific approaches seem to be one of the keys to minimize the potential impacts of climate change and maximize the effects of control and prevention programs in PNG.

## Abbreviations

AIC, Akaike’s Information Criterion; ENSO, El Niño-Southern Oscillation; EMI, El Niño Modoki index; GLM, generalized linear model; IOD, Indian Ocean Dipole; JAMSTEC, Japan Agency for Marine-Earth Science and Technology; NOAA, National Oceanic and Atmospheric Administration; PNG, Papua New Guinea; SAM, Southern Annular Mode; SST, sea surface temperature

## Additional file

Additional file 1: Table S1. The periods of time for the respective local weather and global climate models for each study locations. Table S2: The crude Pearson’s correlations among malaria cases and local weather factors during the study period at each study location. Figure S1: Cross-correlations for malaria cases and local weather factors. Cross-correlations identify the lagged relationships. The correlograms shows the correlations between malaria cases at time *t* and local weather at lag time *t* + *k* (i.e., *k* is a lag). Figure S2: Cross-correlations for malaria cases and global climate factors. The associations between malaria cases at time t and global climate indices at time *t* + *k* (i.e., lag). Figure S3: Time series plots for malaria cases, precipitation, and minimum temperature in each region during the study period. Figure S4: Correlation, histogram, and plot matrix for EMII, NINO3.4 Anomaly, DMI and SAM during 1997 – 2008. (PDF 1057 kb)
