# Supplementary material for: Associations between malaria and local and global climate variability in five regions in Papua New Guinea
Source: Trop Med Health. 2016 Aug 4;44:23. doi: 10.1186/s41182-016-0021-x (PMC4972963; doi:10.1186/s41182-016-0021-x)

SUPPLEMENTAL MATERIALS: Associations between malaria and local and global climate variability in five regions in Papua New Guinea

Chisato Imai, Hae-Kwan Cheong, Ho Kim, Yasushi Honda, Jin-Hee Eum, Clara T. Kim, Jin Seob Kim, Yoonhee Kim, Swadhin K. Behera, Mohd Nasir Hassan, Joshua Nealon, Hyenmi Chung, and Masahiro Hashizume

Table S1: The periods of time for the respective local weather and global climate models for each study locations.

Region	Local weather			(missing data)		Global climate		
	start	end	years	Minimum temperature	Precipitation	start	end	years
**East Sepik**	1997	2008	12	1.4%	0.7%	1997	2008	12
**Madang**	1997	2006	10	0.0%	0.0%	1997	2008	12
**Eastern Highlands**	1999	2008	10	13.3%	11.7%	1997	2008	12
**Western**	1998	2007	10	0.8%	4.2%	1998	2008	11
**Port Moresby**	1997	2007	11	0.8%	0.0%	1997	2008	12

Table S2: The crude Pearson's correlations among malaria cases and local weather factors during the study period at each study location.

Region	Malaria	Malaria	Precipitation
	Precipitation	Minimum temperature	Minimum temperature
**East Sepik**	0.06	0.27	0.02
**Madang**	0.24	0.10	0.01
**Eastern Highlands**	0.01	0.28	0.50
**Western**	0.30	0.05	0.25
**Port Moresby**	0.21	0.15	0.23

Figure S1: Cross-correlations for malaria cases and local weather factors. Cross-correlations identify the lagged relationships. The correlograms shows the correlations between malaria cases at time t and local weather at lag time $t + k$ (i.e. k is a lag).

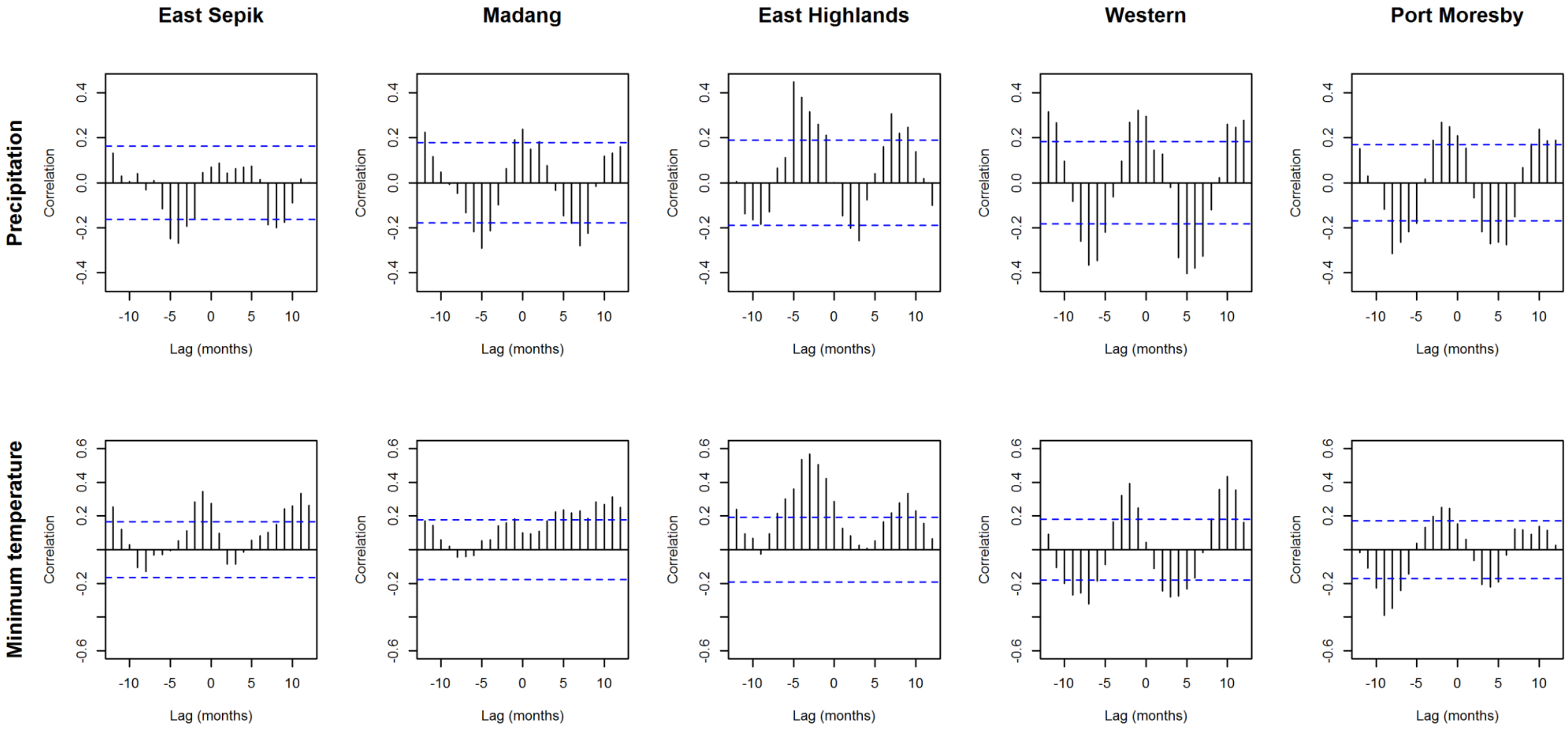

Figure S2: Cross-correlations for malaria cases and global climate factors. The associations between malaria cases at time *t* and global climate indices at time *t* + *k* (i.e. lag).

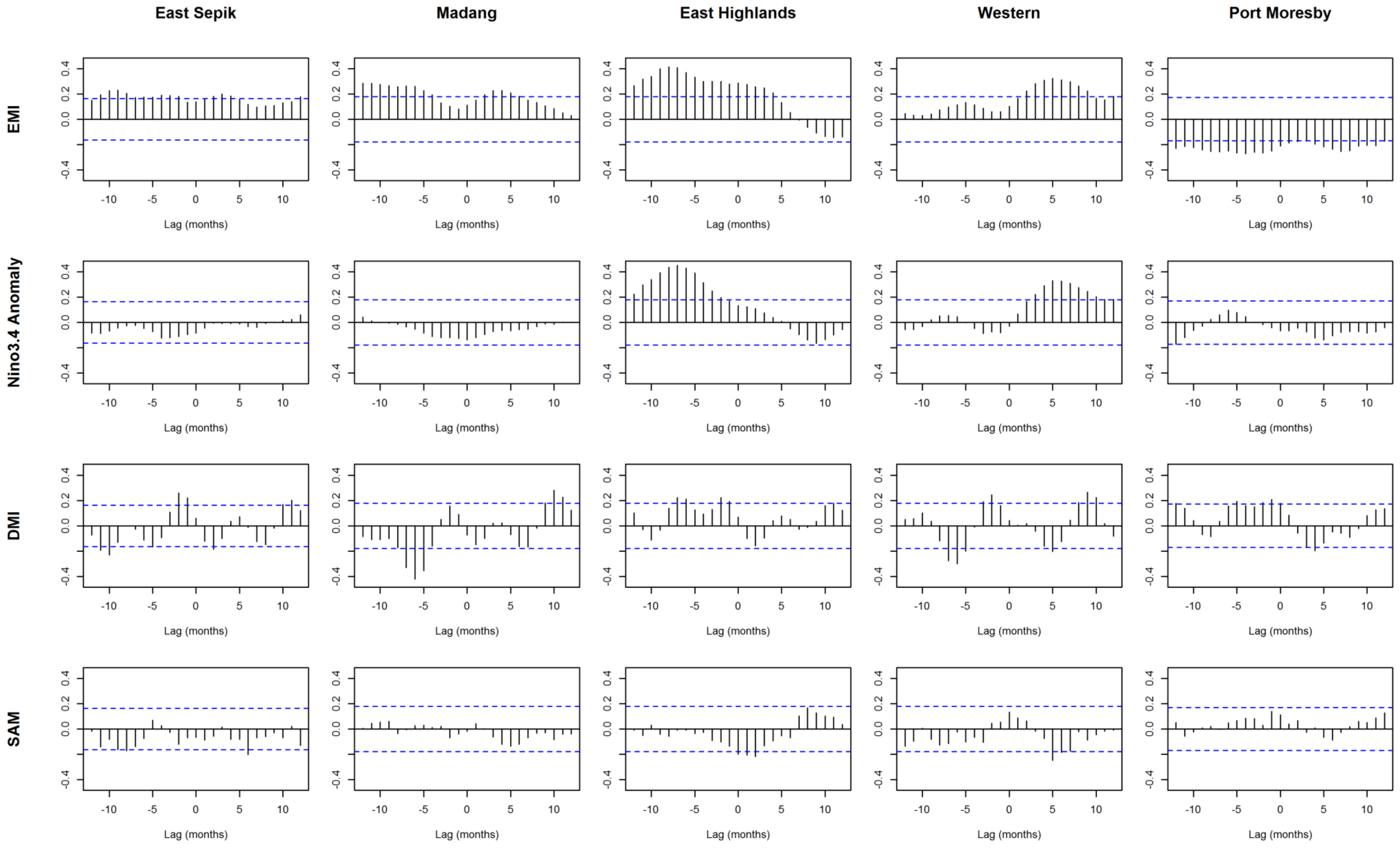

Figure S3: Time series plots for malaria cases, precipitation, and minimum temperature in each region during the study period.

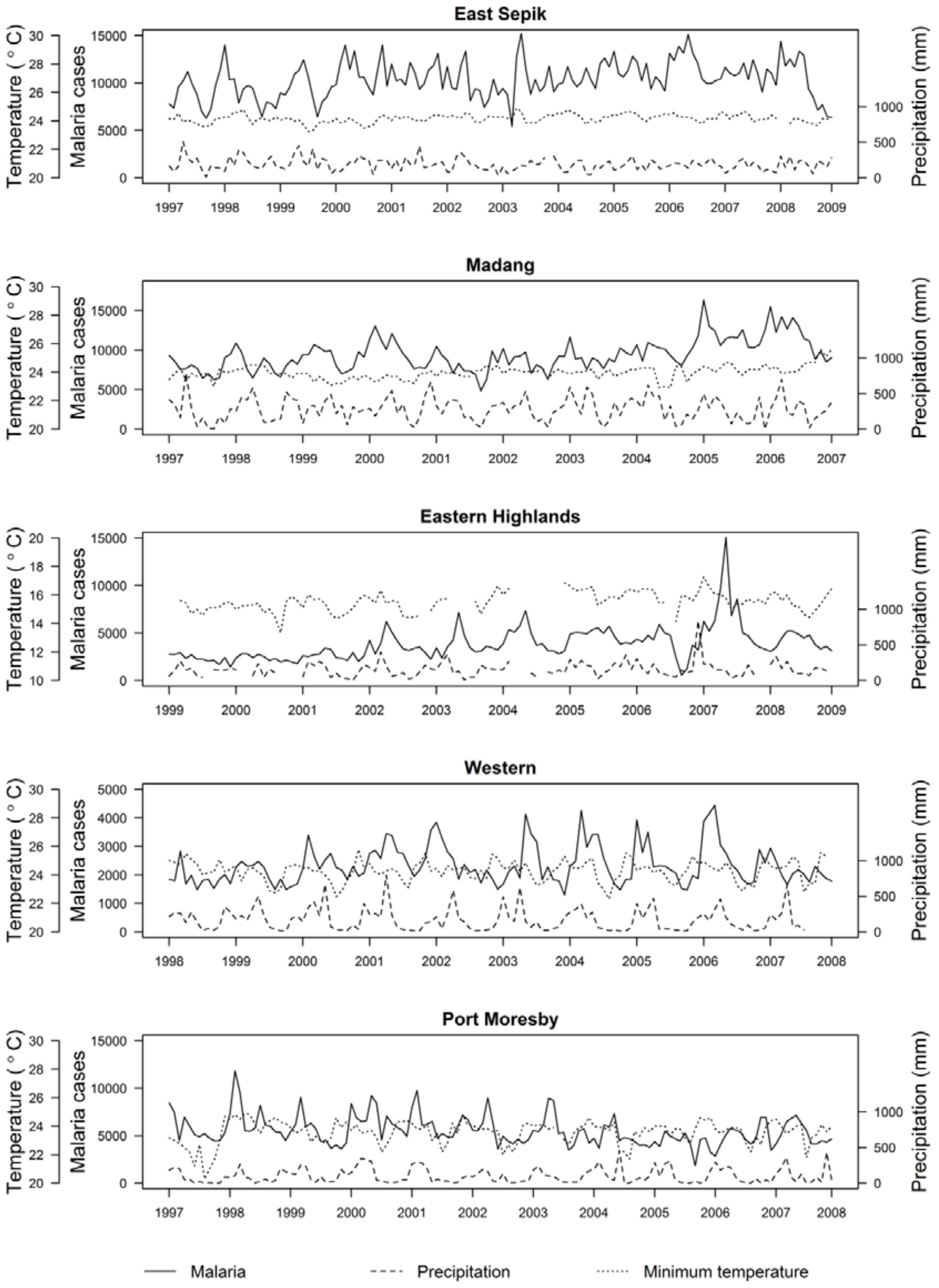

Figure S4: Correlation, histogram, and plot matrix for EMII, NINO3.4 Anomaly, DMI and SAM during 1997 – 2008.

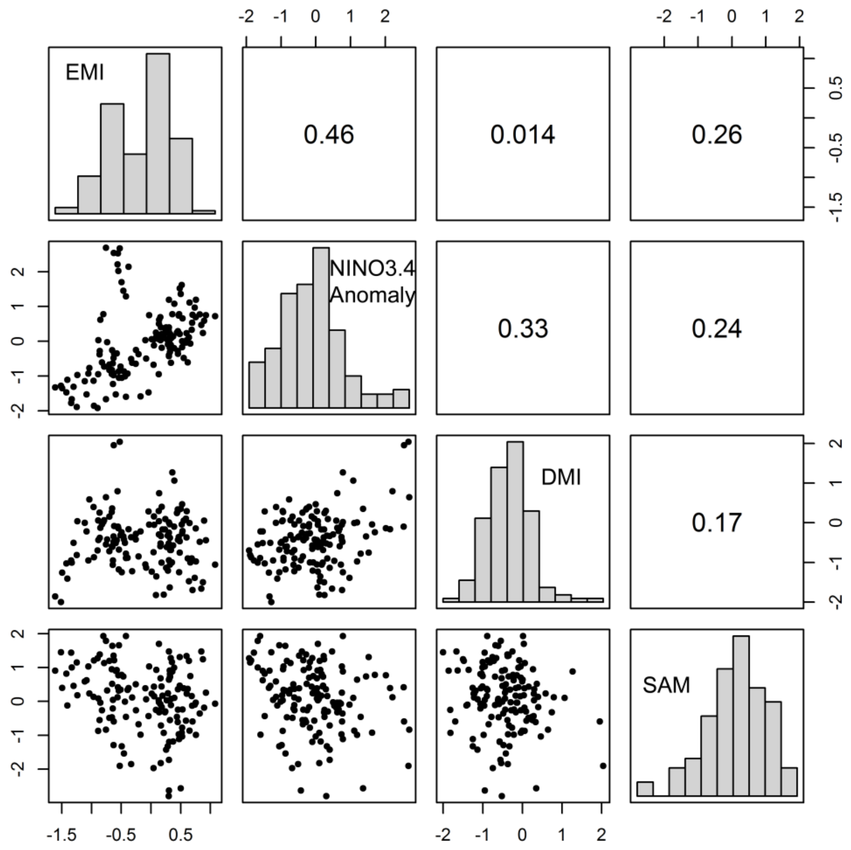

Supplement: Additional file 1: Table S1. — The periods of time for the respective local weather and global climate models for each study locations. Table S2: The crude Pearson’s correlations among malaria cases and local weather factors during the study period at each study location. Figure S1: Cross-correlations for malaria cases and local weather factors. Cross-correlations identify the lagged relationships. The correlograms shows the correlations between malaria cases at time t and local weather at lag time t + k (i.e., k is a lag). Figure S2: Cross-correlations for malaria cases and global climate factors. The associations between malaria cases at time t and global climate indices at time t + k (i.e., lag). Figure S3: Time series plots for malaria cases, precipitation, and minimum temperature in each region during the study period. Figure S4: Correlation, histogram, and plot matrix for EMII, NINO3.4 Anomaly, DMI and SAM during 1997 – 2008. (PDF 1057 kb) [file 41182_2016_21_MOESM1_ESM.pdf]
